# Effect of Mahjong on children's intelligence quotient

**DOI:** 10.3389/fpsyg.2022.934453

**Published:** 2022-09-26

**Authors:** Takefumi Higashijima, Taisuke Akimoto, Katsumi Sakata

**Affiliations:** Department of Neurosurgery, Yokohama City University Medical Center, Yokohama, Japan

**Keywords:** Mahjong, intelligence quotient, brain function, children's Mahjong class, Wechsler Intelligence Scale for Children

## Abstract

This study investigated the effect of Mahjong, which is a table game played by three or four players and involves intellectual activity, on the intelligence quotient (IQ) of children. The participants were children between the age of 6 and 15 years, and their IQ was assessed immediately after enrolling in children's Mahjong classes and 1 year after the enrollment using the Wechsler Intelligence Scale for Children Fourth Edition (WISC-IV). Twenty children were included in the analysis. Their mean age at the time of the initial evaluation was 9 years and 6 months. In addition, we conducted a 1-year post-examination. The change in the IQ of this group was compared to that of a historical control group with a similar age range and test–retest interval. The mean overall full-scale IQ of the 20 children during the initial and post-1-year examinations was 106.05 and 113.75, respectively, and showed a statistically significant increase (*p* < 0.01). Based on the subscale index, the verbal comprehension index (VCI) and processing speed index (PSI) scores both showed a statistically significant increase from 100.6 to 106.75 and from 108.05 to 119.05 (*p* < 0.01), respectively. The PSI of the children included in the analysis showed a statistically significant increase compared to the historical control group. This study suggests that children who participate in Mahjong classes during their childhood have increased PSI scores of WISC-IV.

## Introduction

Heredity is considered to strongly influence intelligence quotient (IQ). The degree to which genes are involved in an individual's characteristics is indicated by the concept of heritability, expressed as 1.0 when the entire effect can be explained by genes. The heritability of intelligence is approximately 0.8 (Sauce and Matzel, 2018), stronger than the heritability of cancer (0.1), mental disorders (0.4) (Athanasiadis et al., 2022), and body mass index (BMI) (0.6) (Elks et al., 2012). The heritability of BMI is higher during childhood, and the influence of environmental factors becomes stronger as the child grows. In contrast, for IQ, the younger the age, the more the individual is influenced by the environment (Fulker et al., 1988). In addition to environmental and genetic factors, IQ is also affected by training (Sauce and Matzel, 2018).

There is no consensus on whether interpersonal table games are correlated with IQ. For chess, the most studied table game regarding its relationship with brain function, some studies have shown that game skill is correlated with processing speed and executive function (Burgoyne et al., 2016). Furthermore, the average IQ of chess players was approximately 120, higher than the population average. In addition, a weak correlation was found between chess performance and IQ (Bilalić et al., 2007). However, Bilalić et al. (2007) reported that the amount of practice was the factor that affected chess skills the most, but not IQ. This finding was consistent with the report by Campitelli and Gobet (2011). However, the relationship between chess and IQ is unclear, and most studies exploring this relationship were cross-sectional. Hence, a causal relationship has not been clearly identified. In comparison, video games have been the subject of many positive studies, indicating that the effects of video games increased intelligence and cognitive function (Stern et al., 2011; Baniqued et al., 2013; Oei and Patterson, 2013). This is not because video games have a stronger impact on intelligence and cognitive function than table games, but rather because video games are easy and quick, and anyone can play them, making intervention studies easier. Moreover, it is difficult to grasp the rules of table games, such as chess, and they require a certain amount of effort and a minimum level of intelligence as a prerequisite. This makes it difficult to conduct prospective studies; therefore, fewer prospective studies have been conducted.

Mahjong, a table game played by three to four players, uses 136 tiles, four each of 34 types. Each player has 13 tiles and has to take one tile from the remaining blocks in a counterclockwise direction and discard one simultaneously. This process is repeated for approximately 70 rounds to complete the hand as quickly as possible. During this process, the blocks and the opponents' hands are not revealed, and the player must make the most advantageous choice based on the information of their own hand and the tiles discarded by the opponents. The information processing is very complex. Artificial intelligence with algorithms comparable to those of top-level players was not developed until 20 years after Deep blue, which was the artificial intelligence for playing chess created by IBM, defeated the chess champion in 1997 (Campbell et al., 2002; Li et al., 2020). In Mahjong, players are provided with the opportunity to draw new tiles in a counterclockwise direction. However, the order may not turn out as planned, based on the act of “calling,” which allows the player to take the tiles discarded by the opponent. The players not only have to figure out the best move but also have to anticipate the opponent's moves. However, the information to do so is not disclosed, and one has to use the opponent's gestures and facial expressions as clues to guess their moves. Such complexity makes the creation of algorithms for artificial intelligence difficult.

Recently, the effects of playing Mahjong on health, particularly on brain function, have been studied (Table 1). Mahjong has been shown to prevent depression in older adults (Tang et al., 2021), improve cognitive performance, such as verbal memory in patients with mild dementia (Cheng et al., 2006, 2014; Qiu et al., 2019; Zhou et al., 2020; Ding et al., 2022), and improve higher brain functions, such as increased executive function (Zhang et al., 2020). Based on this improvement in executive function, Zang et al. concluded that Mahjong improved higher brain function by activating frontal lobe functions, such as concentration and anticipation. Fujimori et al. (2015) measured the oxygen consumption of the brain while playing Mahjong and reported that the activity of the temporal and parietal lobes of the dominant hemisphere centered on the temporal and parietal lobes. Increased blood flow in these areas has been associated with increased intelligence, particularly language comprehension and processing speed indices (Kazumata et al., 2019; Sandvik et al., 2020). These findings suggest that continued participation in Mahjong may have an effect on IQ.

**Table 1:** List of previous literature studies on Mahjong.

**Author**	**Year**	**Purpose**	**Subject**	**Study design**	**Conclusion**
Cheng et al.	2006	Cognitive function	Elderly people with dimentia twice a week (*n* = 33) Four times a week (*n* = 29)	Randomized controlled Mahjong training for 16 weeks	Improved cognitive symptoms in both groups
Saito et al.	2006	Brain activity of Mahjong expert	Mahjong expert (*n* = 8) Healthy volunteers (*n* = 12)	Cross-sectional	playing while actually touching the Mahjong tiles may alter the cross-modal response of the tactile and visual cortices.
Fujimori et al.	2015	Brain activity	Healthy men (*n* = 14)	Cross over trial Measured oxygenated hemoglobin concentration during Mahjong	Wernicke's area and visual cortex were more activated than control.
Cheng et al.	2014	Cognitive function	Elderly people with dementia (*n* = 110)	Mahjong group (*n* = 36)	Mahjong gradually improved global functioning and relieved symptoms of dementia.
				Tai chi group (*n* = 39)	
				Hand craft group (*n* = 35)	
				Randomized controlled Mahjong training for 12 weeks	
Tsang et al.	2016	Eye-hand coordination	Elderly people who played Mahjong (*n* = 21) did not play Mahjong (*n* = 20)	Cross-sectional	People who play Mahjong have better eye-hand coordination.
Qiu et al.	2019	Cognitive function	Elderly healthy people (*n* = 4,839)	Prospective cohort For 16 years	Playing cards or Mahjong were associated with a decreased risk of cognitive impairment.
Zhou et al.	2020	Cognitive function	Adult people (Age ≥ 45, *n* = 7,973)	Fixed effect analysis	Playing Mahjong and using the internet were associated with improved memory
Zang et al.	2020	Cognitive function	Elderly people with MCI (*n* = 28) control group (*n* = 28)	Randomized controlled Mahjong training for 12 weeks	Playing Mahjong improved the executive function
Tang et al.	2021	Depressive symptoms	Elderly people (*n* = 19,420)	Cross-sectional	Playing cards or Mahjong were protective factors of depressive symptoms
Ding et al.	2022	Cognitive function	Elderly people with MCI (*n* = 187) without MCI (*n* = 489)	Cross-sectional	Years of Mahjong playing were associated with reduced odds of having MCI

MCI, Mild cognitive impairment.

The purpose of this study is to investigate the effects of Mahjong on IQ. Most previous studies on table games and IQ have been cross-sectional ones, making it difficult to infer the causal relationship. In addition, most studies on Mahjong have focused on older adults, and the effects on children are unknown. Furthermore, the effects of environment and training on IQ are likely to be more pronounced in children with lower genetic susceptibility. Hence, we prospectively observed and verified whether intelligence increased in children who began learning Mahjong.

## Materials and methods

### Participants

The participants were 35 children, who were Mahjong beginners, aged between 6 and 15 years, and were enrolled in the Neuron Children's Mahjong Class, Oimachi School, between January 2020 and January 2021. Those who had experience in playing Mahjong were excluded from this study. However, those assigned to the beginners' class who had not learned the rules of Mahjong and could not progress by themselves were included. IQ evaluations were conducted at the time of enrollment in the Mahjong class and after 1 year. For acquiring the participants' background information, we also conducted interviews about other after-school activities, playing video games, having siblings, and having family members who play Mahjong (Table 2).

**Table 2:** Clinical manifestations and life background.

	**Mahjong group**	**Historical control group**
*n*	20	43
Male: female	11:09	18:25
Age at the first examination	9.54 ± 2.47	7.77 ± 1.90
Test-retest interval	12.45 ± 1.09	10.88 ± 1.22
Only child	3	N/A
Sibling participation in the Mahjong class	8 (4 pairs)	N/A
Other family members who can play Mahjong	7	N/A
Playing video games (including smart phone games)	15	N/A
**After-school activities**		
Athletic club	13	N/A
Cram school	14	N/A
Music class	4	N/A
Multiple after-school activities	7	N/A

N/A, not applicable.

### Children's mahjong class

The Children's Mahjong Class was held two times a month on the first and third Sundays at the Neuron Mahjong School Oimachi in Tokyo. In the first few days, the instructor taught the rules. Three or four children sat around a table and played the game while the instructor provided guidance as needed (Supplementary Figure 1). Mahjong is a four-player game, which requires social skills; specifically, it is characterized by teaching patience and politeness, as well as the importance of making speedy decisions and gathering information. In Mahjong, one's action time is simply one-fourth of the game time, and the remaining three-fourths of the time tends to be spent waiting for the opponent's moves. It is especially difficult for young children to sit idle for a long time; they tend to move from their seats as soon as they lose interest, thereby interrupting the game. For this reason, we attempted to keep the game running smoothly by reducing the time of one's turn and reducing the waiting time for the opponent. We also instructed the players to gather information during the waiting time, analyze it, and decide their strategy in advance. Four games were played from 10:30 to 15:00, two in the morning and two in the afternoon with a 30-min lunch break in between and a time limit of 1 h per game. When students reached the intermediate class, they played Mahjong for free. In such cases, opponents were assigned to the same table with people of varying abilities as much as possible. This naturally creates a situation where those with more ability teach those with less ability; thus, the children teach each other.

### Measurement of intelligence quotient

**Figure 1:**
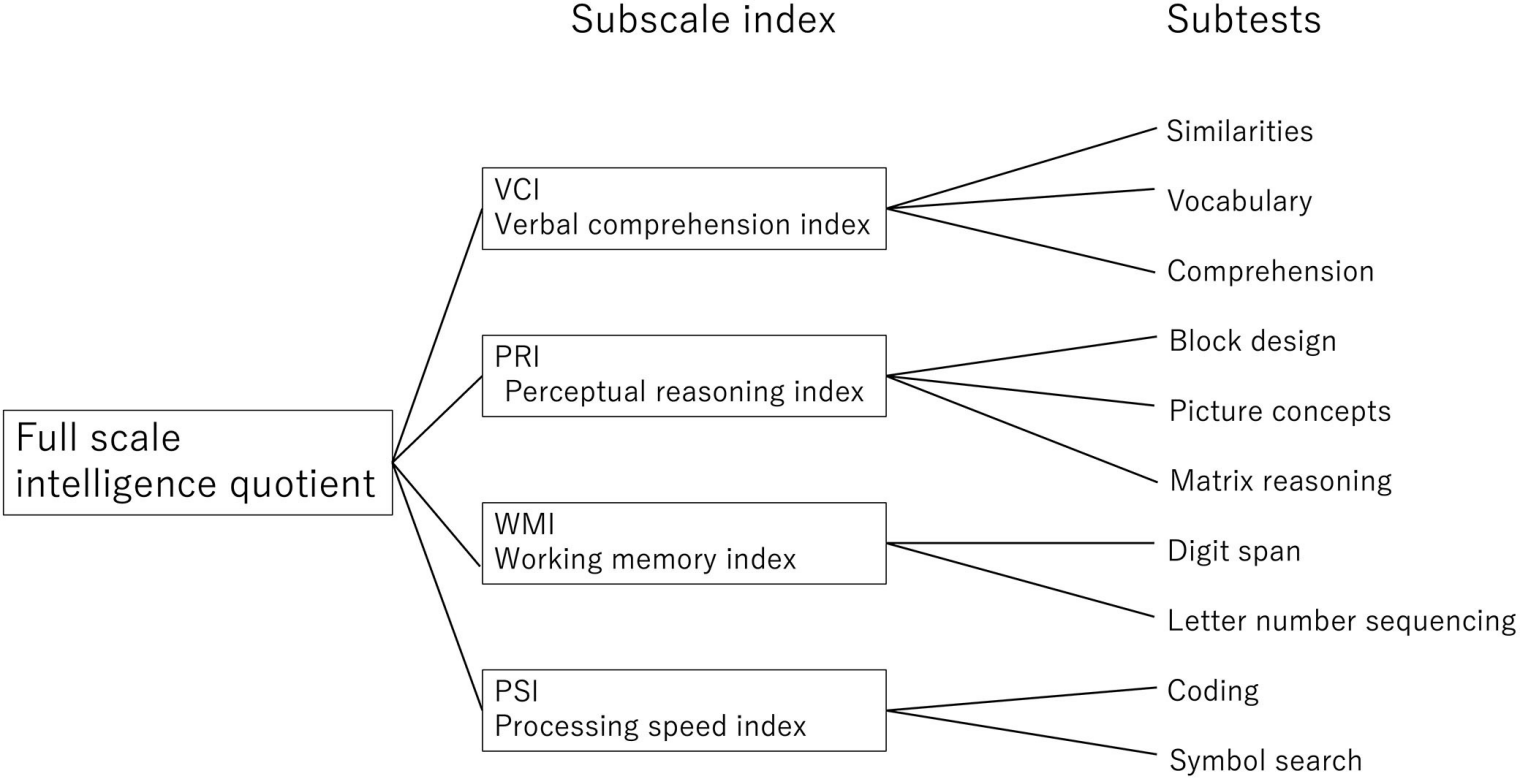
Composition of WISC-IV. The full-test intelligence quotient with the WISC-IV consists of four subscales and a subtest consisting of 2–3 items each.

The Wechsler Intelligence Scale for Children Fourth Edition (WISC-IV) was used to measure the children's IQ. WISC was created in the United States and has been adapted for widespread use in different cultures around the world. It is not just a translation, but a development that takes into account language and culture, making it one of the most adapted tests in the world (Grégoire et al., 2008). Moreover, the WISC 3rd Edition has been used in 12 countries, including Japan and the U.S., to examine cross-cultural differences in intelligence testing, and results showed that the differences in intelligence quotients between cultures were insignificant (Georgas et al., 2003). The WISC-IQ is adjusted to place 50% of the population in the 90–109 range and has been revised every few years. The full-scale IQ (FSIQ), commonly referred to as IQ, is obtained by adding the scores of four subscales: verbal comprehension index (VCI), perceptual reasoning index (PRI), working memory index (WMI), and processing speed index (PRI). Each subscale index is calculated by adding the scores of two or three core subtests: VCI, similarity, vocabulary, and comprehension; PRI, block design, picture concepts, and matrix reasoning; WMI, digit span and letter-number sequencing; and PSI, sign and symbol search (Figure 1). Since the raw scores from each core subtest are adjusted for age to calculate the index score, if the raw score is the same as the previous year, the IQ will decrease. In this study, the scale was administered by a doctor who attended a training course organized by Nihongo Bunka Kagakusha, the publisher of the Japanese version of the WISC, and was qualified to administer the test. All the children were able to complete the core subtests, and no supplemental subtests were substituted.

### Historical control group

In the present study, it was difficult to obtain the cooperation of a control group; therefore, we used a historical control group to compare the effects. The WISC has a rigorous testing methodology; it can be compared with historical controls (Ryan et al., 1994). However, WISC-IV has been shown to increase with shorter test–retest periods due to practice effects (Estevis et al., 2012). To rule out this practice effect, we used data from Ryan et al. (2010) instead of the standardized Japanese WISC4 data in this study. The data from this literature was acceptable as a standard sample of WISC-IV scores over time because the participants were children without learning disabilities or intellectual disabilities, and the test–retest interval confirmed that there was little to no training effect (Table 2).

### Procedure

**Figure 2:**
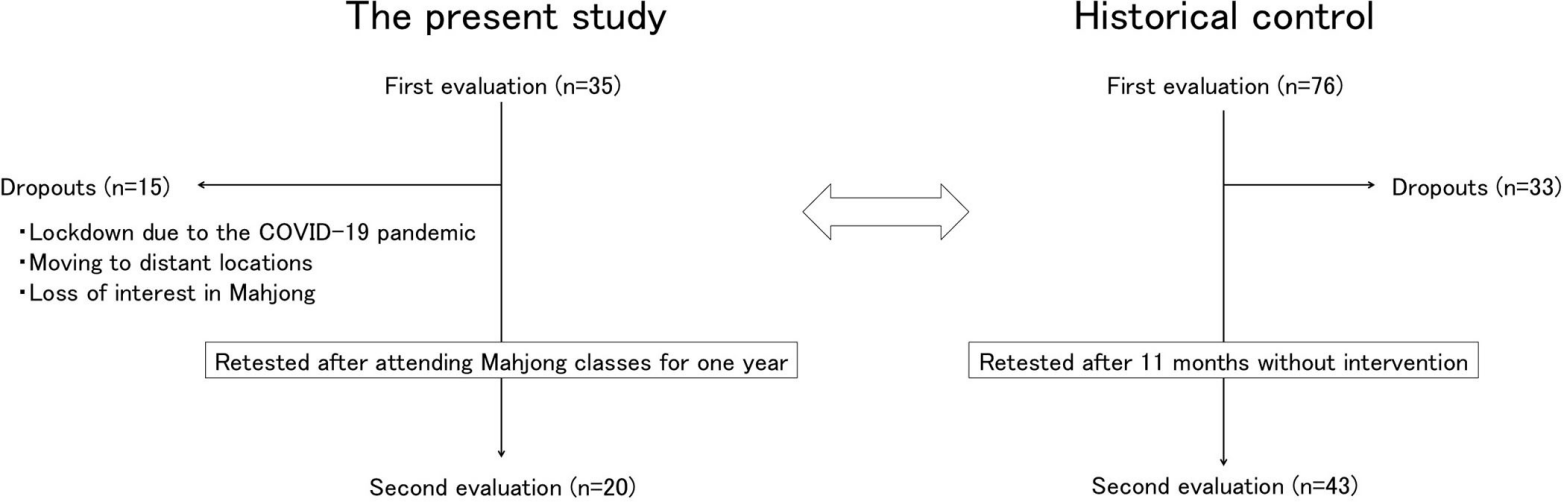
Procedures. An initial evaluation was conducted on 35 patients, but 15 dropped out and 20 were able to complete the second evaluation. The change in intelligence quotient of these 20 participants was compared with that of the historical control group.

Initial IQ evaluation was conducted on 35 children. Fifteen of these children dropped out, and 20 children were evaluated 1 year later; the change in the IQ of the participants was compared to that of the historical control group (Figure 2).

### Statistical analysis

R (version 3.1.3; R Foundation for Statistical Computing, Vienna, Austria; URL http://www.R-project.org/) was used for statistical processing. Fisher's exact test was used for the analysis of binary variables. For between-group comparisons of continuous variables, a *t*-test statistics was conducted for variables following a normal distribution and the Wilcoxon rank sum test for variables not following a normal distribution. For comparisons over time, a paired *t*-test was conducted. A two-way ANOVA was conducted for comparison with the historical control group using Prism 6 (version 6.0 g; GraphPad Software, Inc, San Diego, USA; URL https://www.graphpad.com).

### Ethical considerations

This study was approved by the Ethical Review Committee of Yokohama City University (Ethical Review No.: B201100040). Informed verbal and written consent to participate in the study was obtained from all the children and their parents, respectively. This study was conducted in accordance with the code of ethics set by the Declaration of Helsinki and its future amendments or comparable standards.

## Results

The mean age of the 35 participants who underwent the initial evaluation was 9 years 4 months ± 2 years 2 months (boys = 23; girls = 12). The FSIQ mean was 107.29 ± 13.08, and the mean scores for the subscale indices were as follows: VCI = 105.6 ± 15.02, PRI = 104.89 ± 12.01, WMI = 103.00 ± 16.40, and PSI = 105.91 ± 12.92. Of the participants, 15 stopped coming to the school after the initial evaluation, and 20 (11 boys and 9 girls) continued to attend the Mahjong classes even after 1 year. The average FSIQ of the 15 children who dropped out was 108.93 ± 13.23; the main reasons for dropping out were lockdown due to the COVID-19 pandemic, moving to distant locations, and loss of interest in Mahjong.

At the time of the first evaluation, the mean age of the 20 students who continued to attend was 9 years and 6 months ± 2 years and 6 months (range: 6 years and 2 months to 14 years and 3 months). Their mean FSIQ at the first examination was 106.05 ± 13.17, and there was no difference with the children who dropped out (*p* = 0.53); the mean scores for the subscale indices for these 20 children were as follows: VCI = 100.60 ± 11.77, PRI = 105.80 ± 12.12, WMI = 101.90 ± 15.53, and PSI = 108.05 ± 12.94.

During the second evaluation, their mean age was 10 years and 7 months, and the mean interval between the tests was 12.45 months (range: 11–15 months). The mean number of times the children participated in the Mahjong class by the second evaluation was 16.55 ± 5.30 (4–22). Most students (17/20) attended at least 12 Mahjong classes (at least once a month). In addition, due to the COVID-19 pandemic, few had engaged in new after-school activities for more than 6 months. One of the 20 students began attending a cram school, two had joined an athletics club, and one began playing smartphone games, mostly Mahjong. All these factors were small and difficult to process statistically.

**Figure 3:**
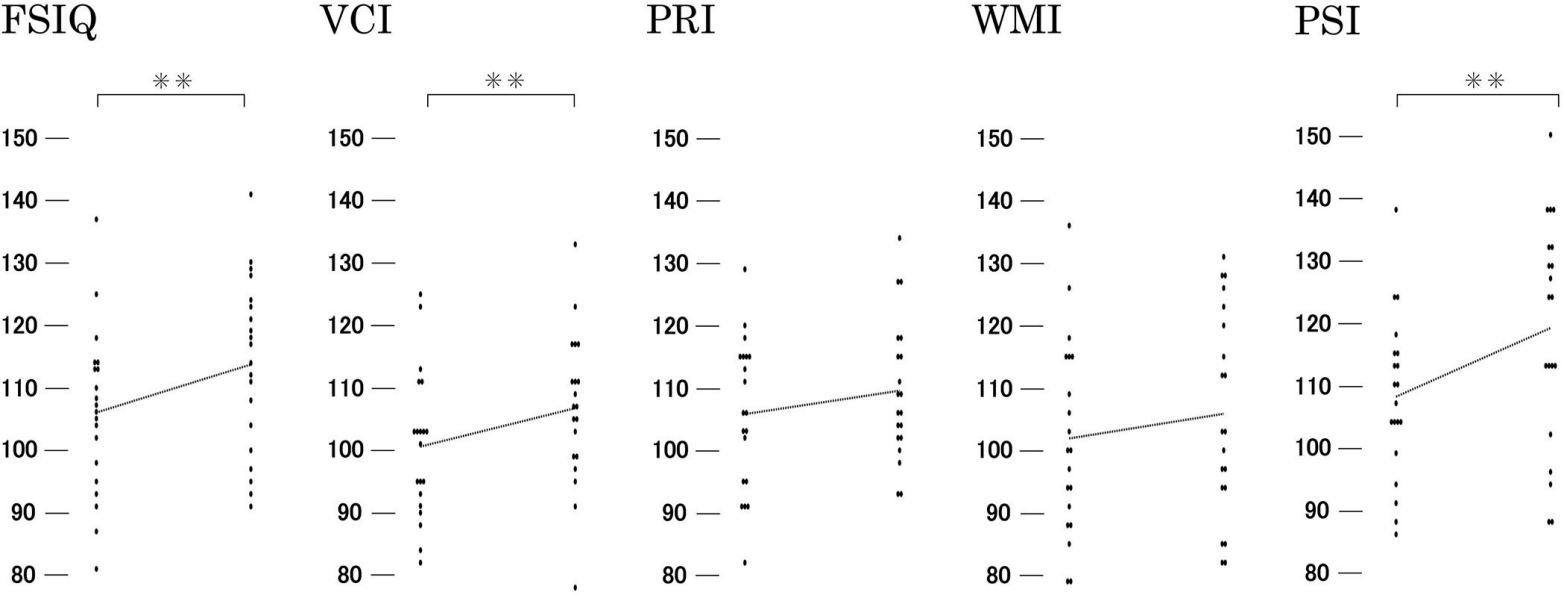
Changes in intelligence quotient. Statistically significant increases were observed in the FSIQ, VCI, and PSI. PRI, Perceptual reasoning index; PSI, Processing speed index; VCI, Verbal comprehension index; WMI, Working memory index; FSIQ, Full-scale intelligence quotient. * *p* ≤ 0.01.

The mean FSIQ at the second evaluation was 113.75 ± 13.86; the mean scores for the subscale indices were as follows: VCI = 106.75 ± 12.09, PRI = 109.55 ± 11.20, WMI = 105.85 ± 16.849, and PSI = 119.05 ± 18.07. There was a statistically significant increase in FSIQ, VCI, and PSI scores (*p* < 0.01) compared to the first evaluation (Figure 3).

There were no significant differences between the participants and the historical control group regarding the changes in FSIQ and VCI; only changes in PSI and symbol search showed statistically significant differences (Table 3).

**Table 3:** Changes in the score of intelligence test.

	**Mahjong group (*****n*** = **20)**				**Historical control group (*****n*** = **43)**				
	**First examination**		**Second examination**		**First examination**		**Second examination**		
	**Mean**	**SD**	**Mean**	**SD**	**Mean**	**SD**	**Mean**	**SD**	***p*-value**
Full-scale IQ	106.05	13.17	113.75	13.86	111.63	10.71	113.26	10.4	0.07
**Subscale index**									
Verbal comprehension index	100.6	11.77	106.75	12.09	107.84	14.69	108.09	13.53	0.14
Perceptual reasoning index	105.8	12.12	109.55	11.2	112.56	11.58	112.98	11.43	0.52
Working memory index	101.9	15.53	105.85	16.849	106.67	14.22	108.72	14.24	0.65
Processing speed index	108.05	12.94	119.05	18.07	107.4	13.01	109.86	11.72	**0.02**
**Subtest**									
Similarities	10.8	1.9	12	2.3	11.16	2.81	11.67	3.23	0.32
Vocabulary	10.13	2.56	10.53	2.61	11.49	2.64	11.47	2.67	0.86
Comprehension	9.47	2.5	11.27	3.28	11.7	3.14	11.47	2.71	0.11
Block design	11.33	3.96	11.53	3.38	11.49	2.64	12.05	2.71	0.97
Picture concepts	9.73	3.24	10.53	2.42	12.16	2.48	12.33	2.34	0.54
Matrix reasoning	12.67	2.79	12.13	2.64	12.37	2.45	11.84	2.6	0.76
Digit span	9.87	2.83	10.2	3.23	10.91	2.84	11.21	2.82	0.92
Letter-number sequencing	10.73	3.73	11.67	3.52	11.76	2.72	12.07	2.75	0.55
Coding	11.53	3.04	12.6	3.94	11.23	3.18	11.47	2.67	0.48
Symbol search	12.87	2.64	14.87	3.54	11.26	2.34	11.98	2.33	**0.03**

IQ, Intelligence quotient.

In addition, groups with PSI elevated by 10% or more compared with those without PSI. We examined the factors in the group with elevated PSI and found that only high WMI (>100) at the first examination was a significant factor (*p* = 0.02). Although there were no significant differences, PSI was likely to be elevated in children who were female (Odds ratio 3.9, 95% CI 0.44–55.62) and who were engaged in athletic lessons (Odds ratio 5.11, 95% CI 0.54–76.37) (Table 4).

**Table 4:** Comparison between increased and non-increased groups of PSI.

		**PSI increase**			
		**More than 10%**	**Less than 10%**	**Odds (95% CI)**	***p*-value**
*N*		11	9		
Female		6	2	3.9 (0.44–55.62)	0.2
Age at the first examination		10.10 ± 2.98	9.08 ± 1.98		0.37
Test-retest interval (Median; IQR)		12 (12–12)	13 (12–14)		0.07
Number of children attending the Mahjong class (Median; IQR)		21 (9–21)	16 (13.5–19.5)		0.49
Only child		1	2	2.71 (0.11–184.85)	0.57
Sibling participation		4	4	0.73 (0.08–6.1)	1
Other family members can play Mahjong		4	3	1.14 (0.13–11.13)	1
Playing video games (including smart phone games)		8	7	0.77 (0.05–8.98)	1
**After-school activities**					
Athletic lessons		9	2	5.11 (0.54–76.37)	0.16
Cram school		7	7	0.52 (0.04–5.12)	0.65
Music lesson		3	1	2.88 (0.18–176.62)	0.59
Multiple after-school activities		3	4	0.49 (0.05–4.31)	0.64
**Initial IQ tests** **≥100**					
Full-scale IQ		9	5	3.36 (0.34–50.18)	0.34
Verbal comprehension index		7	4	2.1 (0.26–18.8)	0.65
Perceptual reasoning index		9	5	3.36 (0.34–50.18)	0.34
Working memory index		9	2	13.05 (1.28–236.66)	**0.02**
Processing speed index		9	6	2.16 (0.19–33.41)	0.62

PSI, Processing speed index; IQR, Interquartile range; IQ, Intelligence quotient.

## Discussion

In this study, children who continuously participated in the Mahjong classes for 1 year demonstrated increased IQ scores. By subscale index, the increase in VCI and PSI was large, whereas the increase in PSI was significant compared with the historical control group (*p* = 0.02). In the subtest that defined the PSI, symbol search showed an increase of more than 1 point and was statistically significant compared with the historical control group (*p* = 0.03). The historical control group was from a different country from the mahjong group, which could be an indication of cultural differences. However, as noted earlier, the WISC has been adapted and widely used in different countries, and the differences in scores across cultures are insignificant (Georgas et al., 2003; Grégoire et al., 2008). There is a strong correlation between WISC-III and WISC-IV, and the Japanese and U.S. versions are very similar in their standardized sample data. In addition, the test–retest stability of the Japanese and U.S. versions of the WISC4 standardized sample data is also very similar, and no significant differences in test score changes occur even when there is a cultural difference (Table 5) (Wechsler, 2003, 2010). Takeuchi et al. (2018) evaluated changes in intelligence quotients of Japanese children, in general, using WISC-III. According to these studies, although there is a negative correlation between intelligence quotient and the amount of time spent on the Internet, little change in the intelligence quotient itself is observed. These findings suggest that IQ may be stable over time in standard Japanese children in the absence of special intervention. In the international literature, it is also known that music training (Carioti et al., 2019), video games, and video viewing can increase WISC scores (Soares et al., 2021). However, in the present study, these particular types of training were also rarely observed in a sustained manner during the observation period.

**Table 5:** Japanese WISC IV comparison with United States.

	**Japanese WISC IV comparison with United States**			
	**Test-retest stability**		**Correlations between WISC-IV and III**	
	**Japanese**	**United States**	**Japanese**	**United States**
Full-Scale IQ	0.93	0.93	0.86	0.89
Subscale index				
Verbal comprehension index	0.91	0.93	0.87	0.87
Perceptual reasoning index	0.78	0.89	0.65	0.74
Working memory index	0.82	0.89	0.7	0.72
Processing speed index	0.84	0.86	0.81	0.81
Subtest				
Similarities	0.85	0.86	0.75	0.76
Vocabulary	0.8	0.92	0.72	0.82
Comprehension	0.74	0.82	0.71	0.62
Block design	0.81	0.82	0.75	0.77
Picture concepts	0.63	0.76	N/A	N/A
Matrix reasoning	0.64	0.85	N/A	N/A
Digit span	0.87	0.83	0.75	0.77
Letter-number sequencing	0.67	0.83	N/A	N/A
Coding	0.84	0.84	0.79	0.76
Symbol search	0.74	0.8	0.69	0.67

Note: IQ, Intelligence quotient; WISC, Wechsler Intelligence Scale for Children.

We analyzed the factors and found that two of them may be related. The first is an increase in simple writing speed and the second is an improvement in visual working memory. Among the core subtests in the WISC-IV, only the two tests to define PSI were administered in writing by subject form within a time limit. Mahjong showed an increase in the emphasis on the eyes and hands and improved the reaction speed and accuracy of the hand movements (Tsang et al., 2016). Moreover, Mahjong might enhance the eye and hand coordinated movements, which led to an increase in symbol search scores. In particular, this effect may be synergistic with the fact that the student is learning athletic lessons. Although no significant differences were found in this study, the PSI tended to be particularly elevated in children who were engaged in athletic lessons. Such children had smooth hand movements, to begin with, and the enhanced hand–eye coordination may have increased their writing speed.

As symbol search was performed by visually recognizing shapes, visual working memory was also reflected in the coarse points. All of the working memory measured by the WISC-IV reflects auditory working memory, which is the ability to remember what one hears. There are a few situations in daily life in which action selection is visually dependent. In daily life, most of the instructions and other things that must be remembered are obtained through hearing. Instructions obtained visually are easier to retain in the form of images or notes, and often do not require short-term memory. In Mahjong, auditory information is essentially not needed, and almost all the information needed for action selection is derived from vision. In the Mahjong classes, children are taught to count, check, and memorize the tiles on the table with their eyes so that they can make speedy decisions when it is their turn. It is highly likely that this type of instruction has influenced the increase in visual working memory. Furthermore, in this study, higher initial working memory (>100) was significantly associated with higher PSI. This suggests that latent memory is related to visual working memory. Children with higher initial PSI (>100) were not associated with increasing PSI; however, children with higher initial PSI may have encountered more situations in which they used visual working memory. Moreover, in this study, PSI was more likely to be elevated in women with auditory working memory dominant over the visual in daily life (Voyer et al., 2017). It has been pointed out that children who originally have high IQ tend to learn better, and their IQs, including PSI, are more likely to increase on the second WISC test (Ryan et al., 2010). This is believed to be a training effect, in which taking an intelligence test multiple times increases the likelihood of higher scores; nevertheless, there was no association between the FSIQ and the rate of increased PSI in this study. The basis for increased PSI might be affected by latent memory, but not by learning ability. Research shows that environmental factors and training may be more effective in increasing IQ at a younger age. No correlation was found between age and increased IQ in this study. However, all participants in this study were school-age children, and the age range was too narrow to examine age correlations. Moreover, elevated PSI is known to occur in teenage, and is less likely to occur later in adulthood (Pauls et al., 2013), which may make a difference in studies with adults.

These effects cannot be obtained by playing Mahjong in a game but may only be obtained by actually playing Mahjong with other people. Fujimori et al. reported that increased blood flow in the visual and language areas of the left temporal and parietal lobes was observed while playing Mahjong (Fujimori et al., 2015). This brain activity was not observed during the game of Mahjong; however, it was observed during both the actual Mahjong with and without a conversation. Mahjong experts have also been shown to activate the primary visual cortex when they touch Mahjong tiles, even when blind-folded (Saito et al., 2006). This response did not occur when a non-Mahjong player touched a Mahjong tile, nor did it occur when a Mahjong expert touched a non-Mahjong tile, such as Braille. Thus, playing while actually touching the Mahjong tiles may alter the cross-modal response of the tactile and visual cortices. Such responses of the visual cortex are also involved in the increase of visual working memory. In addition, not just any table game played with people will show this kind of response. It has been suggested that brain activity during table games varies with the amount of information required for the game. In a chess-like one-on-one complete information game, which requires relatively little information, the caudate nucleus is mainly active (Wan et al., 2011, 2012). This region is used for computational processing, pattern recognition, and other processes similar to deep learning by artificial intelligence. Such brain activity seems to be more involved in learning effects than in visual activity or language functions. Similarly, Burgoyne et al. (2016) reported that numerical ability was more strongly correlated with chess skills than verbal and visuospatial abilities. In a comparison of skilled and novice Buduk players, changes were observed in the nucleus accumbens and amygdala of the skilled players (Jung et al., 2013). The nucleus accumbens acts as a part of the reward system and influences the prediction and expectation, as well as pleasure, when a reward is obtained. The amygdala (a part of the limbic system) also plays a major role in emotion, mainly in reading facial expressions. These findings suggest that in Buduk, where there is more than adequate information for pattern recognition, it is more important to read the thoughts and facial expressions of the opponent and predict their moves than in chess. Conversely, autism spectrum disorders are representative of disorders in which amygdala dysfunction is noticed (Yang et al., 2021). There are various types of autism spectrum disorders, including ADHD and LD, but all types are known to have reduced PSI (Sherman et al., 2012). Research suggests that the amygdala may also be involved in gaming addiction, which has become a problem in recent years. In gaming addiction, a decrease in PSI is also observed, along with VCI and FSIQ, which is contradictory to the neuropsychological test in this study (Jang et al., 2021). Furthermore, PSI may not reflect only the activity of these localized areas. PSI is decreased in epilepsy (Sherman et al., 2012) and head trauma (Rackley et al., 2012), as well as in autism. In these disorders, the PSI is decreased independent of the location of the brain lesion. Therefore, PSI has recently been considered to reflect both local brain functions and extensive brain networks. The extremely varied information processing, including non-verbal communication, enhances the network throughout the brain, leading to an increase in PSI.

## Conclusion

This study showed that continuous participation in Mahjong classes improved PSI. This was significant when compared to the historical control group. The brain network may have been strengthened by the variety of information processing tasks in Mahjong.

## Limitations

In this study, the participants were volunteers, and the sample size for the test was small due to the prevalence of COVID-19. The statistical test power was therefore limited. In addition, it was impossible to conduct a second test using a group that did not participate in the Mahjong class as a control, and the historical control was used as a standardized sample for comparison. This control group had a different cultural background, and the details of their education were unclear. Therefore, comparisons should be made with caution. Comparison with the prior literature, which is difficult to compare, showed no significant differences in FSIQ and VCI, but also confirmed that FSIQ and VCI were significantly increased. Further studies with larger sample sizes and control groups are needed to clarify that Mahjong helps improve IQ. In addition, the Mahjong classes themselves were only held once or twice a month. However, the frequency of playing Mahjong outside the class, that is., the time spent playing Mahjong with friends and family, could not be quantified. We cannot eliminate the possibility that this is an effect of learning Mahjong by attending Mahjong classes, rather than an effect of the Mahjong classes.

## Data availability statement

The original contributions presented in the study are included in the article/Supplementary material, further inquiries can be directed to the corresponding author.

## Ethics statement

The studies involving human participants were reviewed and approved by Ethical Review Committee of Yokohama City University. Written informed consent to participate in this study was provided by the participants' legal guardian/next of kin.

## Author contributions

TH carried out the experiment and wrote the manuscript with support from TA. Both TH and TA contributed to the final version of the manuscript. KS supervised the project. All authors contributed to the article and approved the submitted version.

## Funding

This work was supported by research grants from JSPS Kakenhi (JP20K17976 to TH).

## Conflict of interest

The authors declare that the research was conducted in the absence of any commercial or financial relationships that could be construed as a potential conflict of interest.

## Publisher's note

All claims expressed in this article are solely those of the authors and do not necessarily represent those of their affiliated organizations, or those of the publisher, the editors and the reviewers. Any product that may be evaluated in this article, or claim that may be made by its manufacturer, is not guaranteed or endorsed by the publisher.

## Supplementary material

Supplementary Figure 1 Lesson outline for Children's Mahjong Class. There were three main levels of children's Mahjong classes: immediately after the introductory class, a beginner class, and an intermediate class. When the instructor determined that it is possible to advance to the next level, the student will advance accordingly.
