# Supplementary figures and images for: Effect of Mahjong on children's intelligence quotient
Source: Front Psychol. 2022 Sep 26;13:934453. doi: 10.3389/fpsyg.2022.934453 (PMC9549265; doi:10.3389/fpsyg.2022.934453)

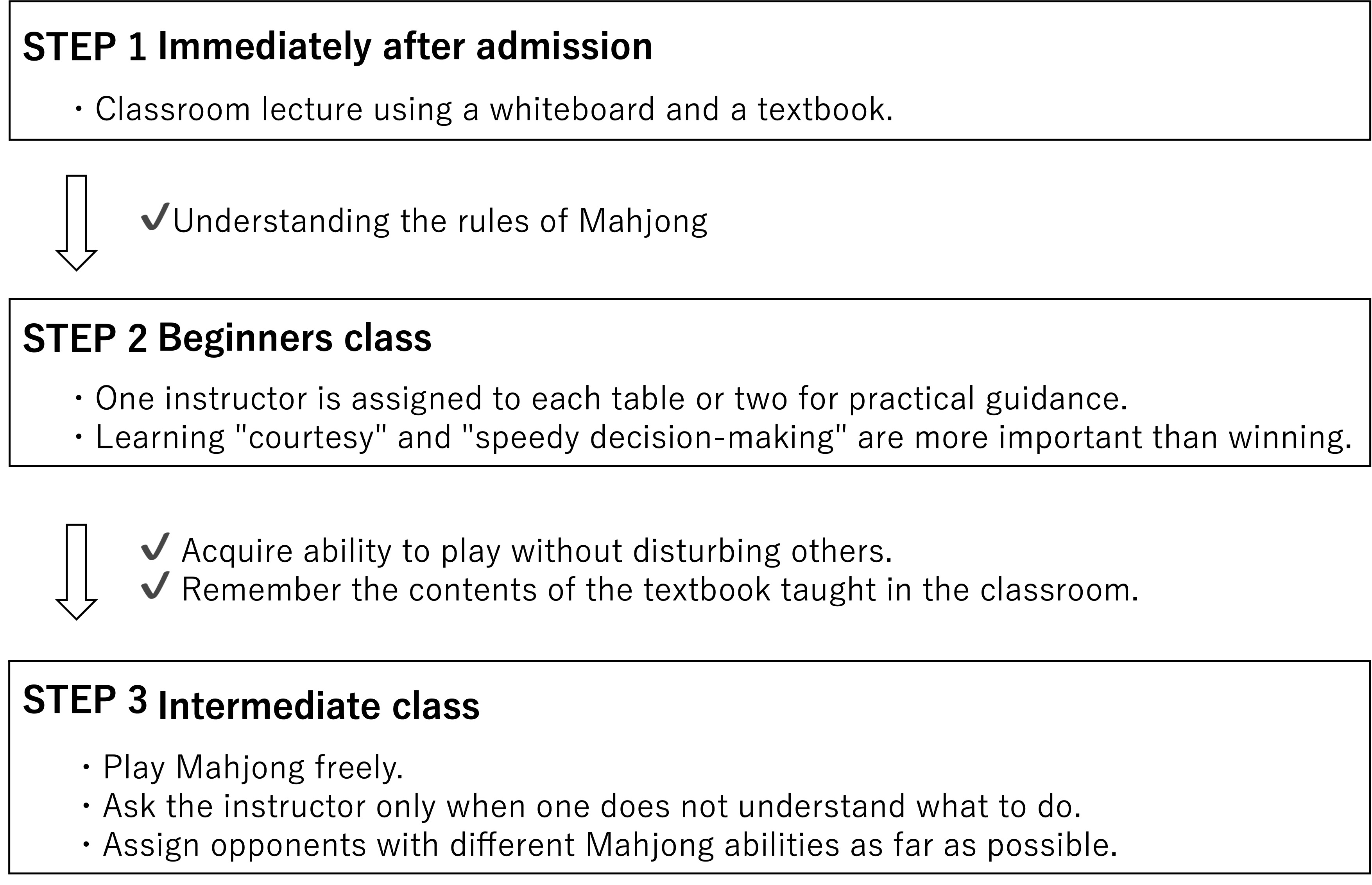

Supplement: Supplementary Figure 1 — Lesson outline for Children's Mahjong Class. There were three main levels of children's Mahjong classes: immediately after the introductory class, a beginner class, and an intermediate class. When the instructor determined that it is possible to advance to the next level, the student will advance accordingly. [file Image_1.jpeg]
